# COVID-19 Vaccine Acceptance, Knowledge, Attitudes and Socio-Demographic Factors of General Population: A Mixed-Methods Study

**DOI:** 10.7759/cureus.82867

**Published:** 2025-04-23

**Authors:** Sindhuja k, Kumari Manjini Jayaram, Molly Mary Thabah

**Affiliations:** 1 Medical Surgical Nursing, College of Nursing, Jawaharlal Institute of Postgraduate Medical Education and Research, Puducherry, IND; 2 Nursing, Jawaharlal Institute of Postgraduate Medical Education and Research, Puducherry, IND; 3 Internal Medicine, Jawaharlal Institute of Postgraduate Medical Education and Research, Puducherry, IND

**Keywords:** acceptance, attitude, covid-19, knowledge, vaccination

## Abstract

Introduction: The COVID-19 vaccine offers the most effective means to control the pandemic. Understanding vaccine acceptance is crucial due to high levels of vaccine hesitancy and relatively low vaccination coverage. The aim of this study is to assess the general population’s knowledge and attitude toward COVID-19 vaccination.

Methods: An explanatory mixed-method approach was used among the general population attending outpatient services at a tertiary care hospital. A total of 369 eligible individuals encountered during the data collection period were included. Their knowledge and attitude regarding COVID-19 vaccination were assessed using a self-structured questionnaire. Additionally, in-depth interviews were conducted to explore reasons for vaccine hesitancy. Statistical analyses included mean with standard deviation (SD) or median with interquartile range (IQR), and correlation coefficients.

Results: Among the 369 participants, 226 (61.2%) demonstrated moderate knowledge, while 241 (65.3%) exhibited a favorable attitude toward the COVID-19 vaccine. A positive correlation between knowledge and attitude was observed (r = 0.114, p = 0.029). Participants perceived the vaccine as a means to prevent infection, build immunity, and ensure safety. Hesitancy stemmed from concerns about side effects, age, health issues, fear of needles, lack of awareness, and media influence.

Conclusion: The primary factor driving vaccine hesitancy was fear of adverse effects following vaccination. Misinformation and fear are significant barriers to achieving global vaccination goals, requiring targeted interventions to enhance awareness and acceptance.

## Introduction

On March 11, 2020, the World Health Organization (WHO) proclaimed a pandemic due to the appearance in 2019 of the coronavirus (COVID-19), which was caused by the SARS-CoV-2 virus [1]. It began in Wuhan, China, at the end of 2019 and quickly spread globally, infecting millions of people in more than 220 countries [2]. The epidemic has severely strained economies, societies, and health-care systems. According to the WHO, there were 529,698,736 confirmed cases of COVID-19 worldwide as of June 1, 2022, and 6,292,503 fatalities [1,2]. On January 27, 2020, the first COVID-19 case was reported in India. As of June 2, 2021, India had reported 43,160,832 confirmed cases and 524,636 deaths overall [3].

One of the most important public health interventions for preventing infectious diseases is vaccination [4]. Having eradicated polio in 2014 and stopped the spread of diseases that can be prevented by vaccination for more than 10 years, India has made significant strides in routine immunization programs [3,4]. To combat the pandemic, however, vaccines had to be developed to combat the rapid spread of COVID-19 [5]. Vaccination is thought to be necessary to completely stop the spread of the disease, especially in highly populated places, even though social isolation and quarantine measures have decreased transmission [6].

Finding safe and efficient vaccines became a top concern as the pandemic spread [7]. A number of vaccine manufacturers had finished phase three studies by the end of 2020, giving them hope that things would return to normal [5-7]. Nine of the 65 vaccines under development as of January 26, 2021, including Oxford-AstraZeneca, BBIBP-CorV, and Sputnik V, had been approved internationally [8]. It was the greatest immunization campaign in the world, with over 68.1 million people vaccinated across 56 nations [9]. Nonetheless, vaccine reluctance has become a major barrier to vaccination efforts [10]. One of the top 10 global health hazards for 2019, according to the WHO, was vaccination reluctance [11]. There are significant differences in vaccine acceptance between nations. In more developed countries, 15-25% of the population has expressed hesitancy, with even higher rates in developing countries [8-11].

According to epidemiologists, the virus might spread to other countries [12]. Consequently, it is essential to develop immunity via either vaccination or spontaneous infection [13]. Pfizer-BioNTech and Moderna’s vaccines have proven to be highly effective, preventing more than 90% and 94.5% of COVID-19 cases, respectively [12,13]. With the launch of Covaxin and Covishield, vaccination efforts have begun in India [14]. Despite the fact that India has rapidly distributed over four million vaccination doses worldwide, questions still surround the effectiveness, safety, and feasibility of such a massive program [15,16].

Researchers have found that vaccine hesitation has several causes, such as misconceptions about vaccinations, possible negative health effects, and worries about the efficacy and safety of vaccines. Inadequate public knowledge of diseases that can be prevented by vaccination and a lack of faith in the medical establishment are further obstacles. Public health initiatives are seriously at risk from misinformation that causes vaccine reluctance [17]. Vaccine hesitancy affects the community as a whole and poses a threat to herd immunity. Vaccine uptake is influenced by a number of factors, including socioeconomic position, education, vaccination cost, past vaccination history, perceived severity of the illness, and faith in medical professionals [18-20]. Vaccination rates during the 2009 H1N1 pandemic ranged widely, from 17% to 67%, underscoring the significance of comprehending and addressing the barriers to vaccine adoption [21-25].

This study’s novelty lies in the fact that the COVID-19 vaccine is a relatively new intervention, likely eliciting a varied response from the public. While some people may embrace vaccination, others may resist, with rumours and false information exacerbating this resistance. In India, where a massive immunization campaign is presently in progress, the purpose of this study is to evaluate the public’s knowledge and attitude about acceptance of the COVID-19 vaccine. Given the size and diversity of India’s population, which makes vaccine administration difficult, this is essential.

Additionally, understanding the reasons behind some healthcare professionals’ own resistance to the vaccine is crucial. Therefore, it is important to ascertain the relationship between knowledge and attitude toward acceptance of the COVID-19 vaccine, establish this relationship and specific demographic variables, and investigate participants’ reluctance to accept the vaccine.

## Materials and methods

**Figure 1:**
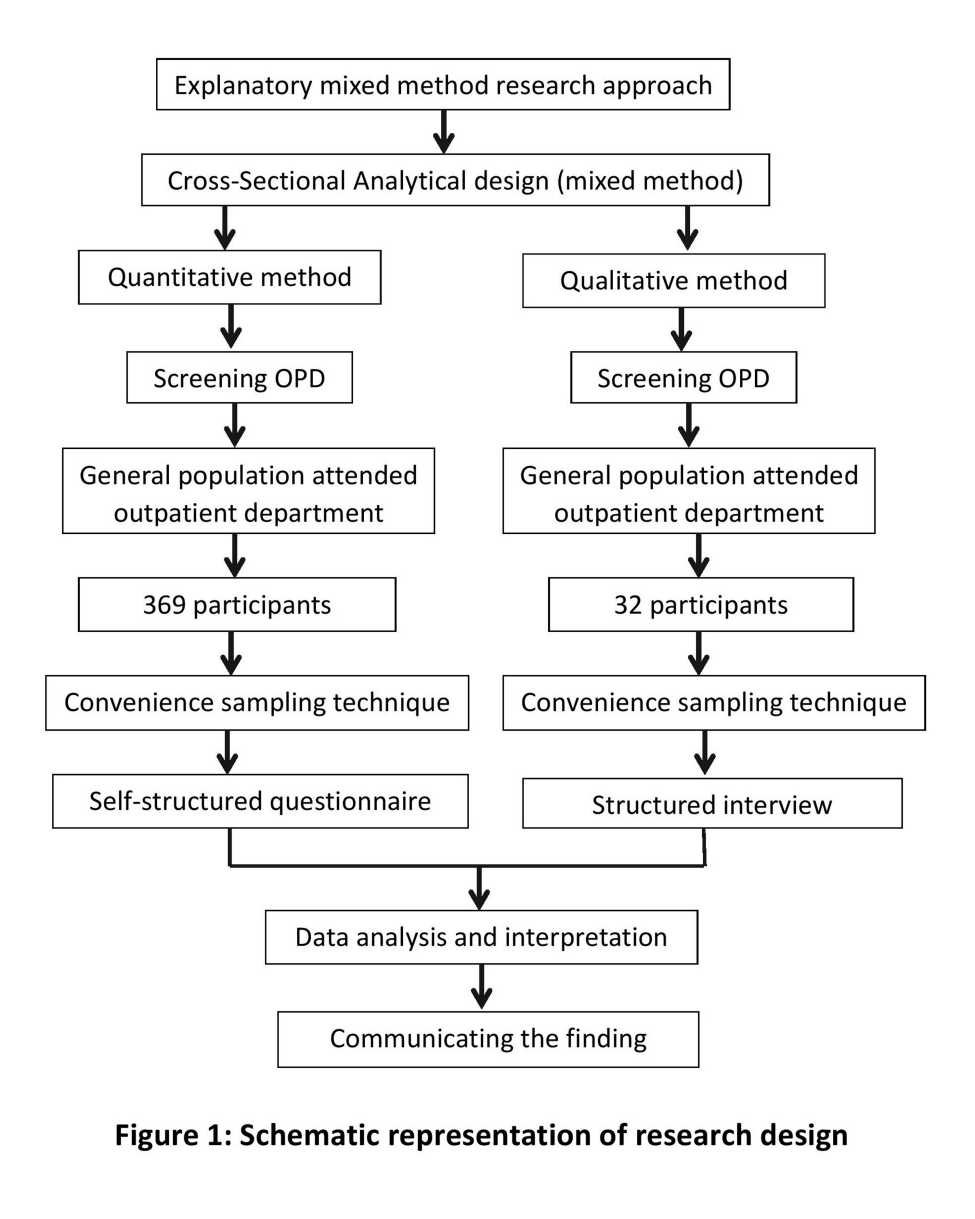
Research design

Developing successful measures to increase vaccination uptake requires a thorough understanding of the information, attitudes, and challenges associated with COVID-19 vaccine acceptance, which has been provided via an explanatory mixed-methods methodology. A thorough investigation of the research issue using both quantitative and qualitative data was made possible by this design. The research design is depicted in Figure 1.

The data collection period was from 2021 to 2022. The study was conducted at a tertiary care teaching and research institute located in South India. Data collection took place at the screening outpatient department (OPD).

The inclusion criteria were people above 18 years of age attending the screening OPD during data collection who could understand either English or Tamil. The exclusion criteria were pregnant and lactating women, individuals with active symptoms of COVID-19, and people with hearing and speech impairments.

The study sample size was 369 participants. This calculation was based on an anticipated 40% prevalence (as per the statistician's suggestion, 40% was considered for prevalence was anticipated) of COVID-19 vaccine knowledge among people aged 18-60 years, with 5% absolute precision and a 5% allowable margin of error. Convenience sampling was used to recruit the participants.

After obtaining their informed consent, data were collected using an interview technique in their native tongue. Sociodemographic information, including age, marital status, gender, educational attainment, residence, occupation, religion, information source, COVID-19 exposure, comorbidity, and number of COVID-19 vaccine doses received, was gathered quantitatively using a self-structured questionnaire. Eight questions with three alternative answers made up the COVID-19 vaccination knowledge questionnaire, which assessed participants’ understanding of COVID-19 vaccine acceptance. One point was given for each correct response. The scores fell into three categories: inadequate knowledge (1-3, < 50%), moderate knowledge (4-6, 50-75%), and adequate knowledge (7-8, 76-100%). Using perceptions across five points to gauge opinions on the adoption of the COVID-19 vaccination, the Likert scale had 14 items with the following responses: strongly disagree (1), disagree (2), neutral (3), agree (4), and highly agree (5). Unfavorable attitude scores ranged from 14-35 (≤ 50%), neutral attitude scores ranged from 36-53 (50-75%), and favorable attitude scores ranged from 54-70 (76-100%). The researcher created the questionnaire based on expert recommendations and an assessment of pertinent literature. Experts in the field then verified it.

Qualitative data gathered by an in-depth interview guide with open-ended questions was used to explore participants’ opinions about COVID-19 vaccination and reasons for vaccine hesitancy. Interviews were conducted until data saturation was achieved. Approval from the Nursing Research Monitoring Committee (No. JIP/CON/NRMC/M.Sc./2020/MSN/7, dated 20.05.2021) and the Institute Ethical Committee (No. JIP/CON/IEC/M.Sc./2020/MSN/7, dated 20.10.2021) was obtained before data collection.

Statistical analysis

Quantitative data

Quantitative data were analyzed using IBM SPSS Statistics version 26 (IBM Corp., Armonk, NY). Descriptive statistics such as frequencies, means, percentages, and standard deviations were used to summarize sociodemographic characteristics, knowledge, and attitude scores. The relationship between knowledge and attitude scores was evaluated using either Spearman’s rank correlation coefficient or Pearson’s product-moment correlation coefficient, depending on the distribution of the data.

Qualitative data

Verbatim transcriptions of recorded interviews were made. To evaluate the transcripts, a deductive coding approach was used, and codes, categories, themes, and subthemes were found. Two researchers coded the transcripts separately to guarantee rigor, and they debated any differences until they came to an agreement. The identified themes and sub-themes were represented using a conceptual framework.

## Results

The study included 369 participants, with a median age of 28 years (IQR: 22-37 years); among them, 260 (70.5%) were between 18 and 35 years of age. Of these, 260 participants (70.5%) were aged 18-35 years, 322 (87.3%) were female, and 263 (71.3%) were married. Regarding educational background, 138 participants (37.4%) were graduates, while 179 (48.5%) were homemakers. Additionally, 202 participants (54.7%) lived in rural areas, 346 (93.8%) identified as Hindu, and 206 respondents (55.8%) reported television as their most common source of health information. 

In terms of medical history, 309 participants (83.7%) had no comorbidities, 329 (89.2%) had not contracted COVID-19, 322 (87.3%) had received the COVID-19 vaccine, and 241 (65.3%) had completed two doses. The findings indicate a high level of vaccination adoption among participants. Table 1 presents the public’s awareness of COVID-19 vaccination adoption.

Table 1 displays the public’s awareness of COVID-19 vaccination adoption. Knowledge levels and demographic factors such as age, marital status, education, place of residence, and health information sources were shown to be significantly correlated. Age, the source of health information, COVID-19 vaccination status, and the number of doses received were also significantly correlated with opinions. The majority of participants correctly identified the vaccine’s efficacy and purpose, with 334(90.5%) identifying the necessity of two doses and 289(78.3%) acknowledging its role in stopping the spread of COVID-19. Additionally, 237(64.2%) of respondents thought the vaccine worked against both ancient and novel coronavirus strains. Nonetheless, several information gaps were identified, including a lack of clarity regarding the immunization schedule following COVID-19 infection.

**Table 1:** Knowledge regarding COVID-19 vaccine acceptance among the general population

Knowledge-related question	Response	Frequency	Percentage
Which of the following functions are associated with vaccines?	Kill the COVID-19 virus, prevent death	141	38.2
	Develop immunity	228	61.8
Which of the following is COVID-19 vaccine(s)?	DPT, TT	39	10.6
	Covishield, Covaxin	330	89.4
Do you believe the vaccine is effective against the new and old coronavirus strains?	No	132	35.8
	Yes	237	64.2
What is the duration between the first and second doses of the COVID-19 vaccine?	4-5 months, 0-1 months	65	17.6
	1-3 months	304	82.4
In COVID-19-infected people, after how many months should they take the COVID-19 vaccine?	2-6 months	214	58
	1-3 months	155	42
What is the purpose of the COVID-19 vaccine?	Protect from another disease, increase the disease severity	80	21.7
	Prevent the spread of COVID-19 infection	289	78.3
How many COVID-19 vaccine dosages do we have to take?	1-4 doses	35	9.5
	1-2 doses	334	90.5
Can a COVID-19-positive person on treatment get a vaccination against COVID-19?	Yes	150	40.7
	No	219	59.3

The general population’s attitude toward approval of the COVID-19 vaccination is displayed in Table 2.

**Table 2:** General population response on attitude regarding COVID-19 vaccine acceptance * indicates negative statement

Attitude-related question	Strongly Disagree N (%)	Disagree N (%)	Neutral N (%)	Agree N (%)	Strongly agree N (%)
The vaccine is an effective method to prevent and control a disease.	1(0.3)	3(0.8)	17(4.6)	160(43.4)	188(50.9)
COVID-19 vaccination should be mandatory.	12(3.3)	28(7.6)	32(8.7)	152(41.2)	145(39.3)
I will encourage my family members and others to take the vaccine.	3(0.8)	8(2.2)	27(7.3)	151(40.9)	180(48.8)
I feel safe after the COVID-19 vaccination.	3(0.8)	13(3.5)	53(14.4)	179(48.5)	121(32.8)
I do not need the vaccine because I believe in Ayurveda*	5(1.4)	12(3.3)	113(30.6)	188(50.9)	51(13.8)
I am not sure about COVID-19 vaccine efficacy*	8(2.2)	36(9.8)	107(29)	140(37.9)	78(21.1)
Vaccines reduce the risk and complications of COVID-19.	4(1.1)	16(4.3)	69(18.7)	177(48)	103(27.9)
Vaccinated people can stop worrying about COVID-19.*	23(6.2)	46(12.5)	61(16.5)	190(51.5)	49(13.3)
The COVID-19 vaccine is safe even though the vaccine was developed rapidly.	5(1.4)	15(4.1)	79(21.4)	160(43.4)	110(29.8)
I don’t need the vaccine because I heard that people died after receiving the vaccination.*	9(2.4)	28(7.6)	84(22.8)	196(53.1)	52(14.1)
I can choose the COVID-19 vaccine to get	22(6)	38(10.3)	90(24.4)	129(35)	90(24.4)
I don’t trust the vaccination.*	6(1.6)	26(7)	90(24.4)	171(46.3)	76(20.6)
I don’t need the vaccine because I am already infected with COVID-19.*	8(2.2)	14(3.8)	82(22.2)	166(45)	99(26.8)
I need to wear a face mask and maintain social distancing even if I am fully vaccinated.	1(0.3)	1(0.3)	3(0.8)	102(27.6)	262(71)

Table 3 shows the level of knowledge regarding COVID-19 vaccine acceptance among the general population. According to an analysis of participants’ attitudes and knowledge of the COVID-19 vaccine, 115(31.2%) showed adequate knowledge, whereas 226 (61.2%) exhibited moderate understanding.

**Table 3:** Level of knowledge regarding COVID-19 vaccine acceptance among the general population Note: The level of knowledge categorized as the score from 1 to 3 (less than 50%) noted as inadequate knowledge, from 4 to 6 (50-75%) mentioned as moderate knowledge and from 7 to 8 (76-100%) stated as adequate knowledge.

Level of knowledge	Frequency (N)	Percentage (%)
Inadequate knowledge	28	7.6
Moderate knowledge	226	61.2
Adequate knowledge	115	31.2
Total	369	100

Table 4 shows that the majority (n=241; 65.3%) had a favorable attitude toward the COVID-19 vaccine, whereas 124 (33.6%) had a moderate attitude.

**Table 4:** Level of attitude regarding COVID-19 vaccine acceptance among the general population Note: The level of attitude categorized as the score from 14-35 (less than 50%) noted as unfavorable attitude, from 36 to 53 (50-75%) mentioned as neutral attitude and from 54 to 70 (76-100%) stated as favorable attitude.

Level of attitude	Frequency (N)	Percentage (%)
Unfavorable attitude	4	1.1
Moderate attitude	124	33.6
Favorable attitude	241	65.3
Total	369	100.0

Table 5 shows higher knowledge levels marginally linked to more favorable attitudes, according to a small positive correlation found between knowledge and attitude (r = 0.114, p = 0.029).

**Table 5:** Correlation between knowledge and attitude regarding COVID-19 vaccine acceptance among the general population *p<0.05 statistically significant

Variables	Correlation value	Statistical inference
Knowledge and attitude	r = 0.114	*p = 0.029

Qualitative findings from interviews that highlighted public attitudes and reluctance toward the COVID-19 vaccine are shown in Table 6.

**Table 6:** Participants’ opinions about the COVID-19 vaccine

Categories	Codes	Quotes
Vaccine is effective	Prevent infection	(1) Before a COVID-19 infection occurs, a vaccine/medication has to be given to prevent the disease. Then people will not have any problems. (2) Taking the vaccine is good for our family and society. Everyone should take the vaccine; (3) The COVID vaccine is essential. Everyone should take the vaccine; social isolation and masks are necessary. (4) The COVID-19 vaccine is good, it prevents COVID-19 infection
	Develop immunity	(1) The vaccine is safe, develops immunity, and prevents further problems; (2) The vaccine is promising; our body tries adapting to the COVID-19 virus. (3) If we take the COVID vaccine, we will be safe, and we can develop immunity
	Safe	(1) Everyone should take the vaccine and protect themselves. Then only children and elders can be safe. (2) Taking the vaccine is a good thing. Some people’s bodies cannot tolerate vaccines, but we cannot do anything, so everyone should take the COVID-19 vaccine, which is safe. (3) The COVID vaccine is to keep ourselves safe, so we are taking [the vaccine]. (4) Taking the COVID-19 vaccine is excellent and safe
Vaccine is not effective	Protect people	(1) Protect themselves; taking the vaccine is people’s wish, but the government makes a significant decision and spends more money on vaccines for good things. (2) I don’t know, but the government says the COVID vaccine is good; no problem will occur. (3) The COVID-19 vaccine is very safe. People with other diseases, like wheezing or heart problems, should consult a doctor before taking the vaccine. (4) The COVID vaccine is suitable for protecting ourselves, so they are telling us to take an injection. We should take the vaccine.
	Lack of faith in the vaccine	(1) Not sure about whether the vaccine is fake or not; death occurs due to the vaccine. In the village, they fear that anything will happen to them. (2) I think after two doses of vaccinations, people are testing COVID-positive, so taking and not taking vaccines are the same.
	Nonexistence of virus	(1) There is no virus, and also, if any disease occurs, we ourselves are the reason.
	Ayurvedic medicine	(1) If they had a cold, the older generation would take the herbal syrup and become cured, but now we are not drinking it.

The efficiency of the vaccine was praised by participants, who noted that it can prevent infection, build immunity, and guarantee the safety of both children and older adults. Negative views did exist, too, such as skepticism regarding the vaccine’s effectiveness because of post-vaccination infection instances and a predilection for Ayurvedic treatments over contemporary medicine.

Table 7 demonstrates that worries about side effects, such as fever, headaches, and more serious consequences, including heart attacks and death, were the main causes of vaccine hesitation. Participants emphasized the impact of fear and false information spread by the media, especially after negative occurrences had been reported. Hesitancy among some groups was also exacerbated by a lack of knowledge and comprehension regarding the vaccine’s safety and advantages.

**Table 7:** Participants’ hesitancy towards COVID-19 vaccine acceptance and refusal

Categories	Codes	Quotes
Reason for vaccine hesitancy	Side effects	(1) They are afraid that vaccination causes upper arm pain. (2) People are not getting vaccines due to fever and headaches. (3) Some people think vaccines will cause health issues and paralysis, so they are unwilling to take them.
	Age-related concern	(1) Only older people have a fear of getting the vaccine
	Health issues	(1) They don’t know what problem they are having in their body. (2) At first, due to COVID-19, vaccine deaths occurred; people had some health issues, so their bodies could not tolerate the vaccine and they died. But is the vaccine the only reason? We cannot tell
	Fear of death	(1) Some of them are afraid; they are told that vaccines cause death. (2) People fear that vaccines cause heart attacks and death; only vaccinated people have died, so they fear getting the vaccine. 3. Whether anything happens to them or causes any death 4. Some people take the COVID vaccine without fear, but some have a fear of death
	Lack of knowledge	(1) I don’t know much about the COVID vaccine. (2) If anyone has any bodily health issue, and if any unfortunate event happens, people think that the vaccine is the cause; in the village, everyone feels the same about the vaccine. (3) The village and aged people don't have an understanding; they think taking the vaccine causes death 4. People don't know about the COVID-19 vaccine and its function
	Lack of awareness	(1) People don’t have awareness about the COVID-19 vaccine; (2) People get wrong information from their surroundings and fear death; people lack COVID-19 vaccine awareness
	Fear of needles	(1) I fear because the needle is inserted fully; I tried to take the COVID vaccine because the doctor insisted on taking the COVID vaccine, but I saw the needle was inserted, so I didn’t take the vaccine.

## Discussion

The present study was conducted to determine the public’s knowledge and attitudes toward COVID-19. A mixed methods approach was used in the study, which allowed for a thorough comprehension of the topic. In-depth interviews and self-structured questionnaires were used to gather data.

The first objective is to assess the knowledge and attitude on acceptance of the COVID-19 vaccination among the general population. Of the knowledge of the population, 115 (31.2%) had an adequate understanding of the COVID-19 vaccination acceptability, whereas the majority, 226 (61.2%), had moderate knowledge. Findings from research from an online survey conducted across six continents reported that the majority of participants exhibited moderate knowledge regarding COVID-19 vaccines, which aligns with the present study’s findings. [17]. In contrast, studies from England and France observed higher levels of understanding, likely due to differences in public health communication and accessibility of vaccine information [18,19]. Regarding attitudes, 241 (65.3%) of participants in the present study had a positive view of COVID-19 vaccination, which is lower than the rates reported in Italy and Belgium [20,21], but higher than those documented in Egypt and England [22,26]. These discrepancies most likely stem from variances in cultural settings, sociodemographic traits, and the timing of the research with respect to vaccination rollouts.

The second objective is to determine the relationship between attitude and knowledge regarding COVID-19 vaccination acceptance. Knowledge and attitudes about the acceptance of the COVID-19 vaccine were found to be statistically significantly positively correlated (r = 0.114, p = 0.029). The p-value of <0.05 was considered statistically significant. This result bolsters the idea that a more positive attitude toward the vaccine’s acceptability is linked to greater information about it. This finding is consistent with another study showing a positive relationship between attitude and knowledge (r = 0.452) [17].

The third objective is to find out the association between knowledge and attitude on acceptance of the COVID-19 vaccine with selected demographic variables. A number of sociodemographic factors, including age, marital status, education, occupation, place of residence, and health information source, were found to be significantly correlated with knowledge. These results align with those of another study conducted in India that found comparable correlations [26]. This implies that different demographic groups might have differing levels of knowledge and access to information on COVID-19 vaccines. Age, source of health information, COVID-19 vaccination status, and number of doses of the COVID-19 vaccine received were found to significantly correlate with attitudes toward the vaccine. These results suggest that views toward vaccination are shaped by age, information sources, and individual experiences.

The fourth objective is to investigate people’s reluctance to adopt the COVID-19 vaccine. Rich insights into the causes of vaccine hesitancy were revealed by qualitative data gathered from in-depth interviews. The important theme that surfaced is the fear of side effects, which is a common concern, with 196 (53.1%) participants expressing concerns about possible negative consequences, especially death. Quotes such as "They believe that upper arm pain following vaccination is the reason they were afraid" and "Most of them are afraid because of side effects" illustrate this fear. After taking it, you may have pain, fever, and other health problems. This result aligns with a study conducted in the US that revealed comparable issues [23].

Secondly, misinformation and ignorance were clear misconceptions regarding the vaccine’s effectiveness and possible long-term consequences. Quotations such as “I think after two doses of vaccination, people getting COVID positive, so taking and not taking the vaccine are the same” exemplify this point. This emphasizes the necessity of knowledge from reliable sources that are understandable, accurate, and easily accessible. The last one is the lack of trust in the government and the healthcare system. Two obstacles were mistrust of the government and worries about the rapidity of vaccine development.

Overall, this study emphasizes the intricate interactions between variables that affect the uptake of the COVID-19 vaccination. It offers useful information to direct public health initiatives meant to raise immunization rates.

Study limitations

The study was conducted at a single tertiary care teaching and research institute in South India, which may limit the generalizability of the findings to other populations and regions.

Recommendations

Focused public health initiatives ought to address side effects, worries, and dispel myths regarding the effectiveness and safety of vaccines. It is essential to work against false information and foster confidence in authorities and healthcare professionals. The unique concerns of various demographic groups require culturally sensitive and customized communication tactics, and ongoing research is crucial to track vaccine hesitancy and modify interventions as necessary.

Public health programs can work to increase vaccination coverage and ultimately shield people and communities from the persistent threat of COVID-19 by comprehending the factors that influence vaccine uptake and addressing issues that contribute to hesitation.

## Conclusions

Despite the fact that most participants had favourable views and moderate to adequate information regarding the COVID-19 vaccine, vaccine reluctance remained because of false beliefs and concerns about side effects. Improving vaccination uptake and reducing public hesitation need to be addressed through focused awareness efforts and truthful media coverage.
